# Health Tract No. 351

**Published:** 1868-11

**Authors:** 


### HEALTH TRACT NO. 351.

### ONIONS

Are considered by those who have made scientific investigations, to possess an amount of nutriment and healthfulness not to be found in any other under-ground vegetable; besides, there, is a certain stimulant effect which is of itself very agreeable to those who love that odoriferous esculent; never having having eaten one and never calculating to perform such a feat, we speak thus far from the testimony of others skilled in chemical dietetics.

Facts appear to show that however healthful and nutritious the onion may be to persons in good health, and in healthful surroundings, they are most pernicious to sick and well when used in a bad atmosphere, where epidemics, such as cholera and typhoids prevail. An army officer writes to the Scientific American in April 1868.

In the spring of 1849 I was in charge of one hundred men on shipboard, with the cholera among the men. We had onions, which a number of the men ate freely. Those who did so were soon attacked and nearly all died. As soon as I made this discovery their use was forbidden. After mature reflection I came to the conclusion that onions should never be eaten during the prevalence of epidemics for the reason that they absorb the virus and communicate the disease, and that the proper use for them is sliced and placed in the sick room, and replaced with fresh ones every few hours.

It is a well-established fact that onions will extract the poison of snakes; this I personally know. Some kinds of mud will do the same.

After maintaining the foregoing opinion for eighteen years, I have found the following well attested: Onions placed in the room where there is small pox will blister and decompose with great rapidity, not only so but will prevent the spread of the disease. I think as a disinfectant they have no equal when properly used.

Assurances are given that an Onion poultice applied morning, noon and night, for three or four days, will cure a felon sometimes, rendering it unnecessary to lance the finger to the bone; which, by the way, is a certain and speedy cure, giving instantaneous relief to the intolerable pain; yet, some may prefer the Onion poultice. The Botanic name of the Onion, which is of the leek and garlic family, is "Allium Cepa;" its juice, mixed with sugar, is useful in the coughs and colds of children; a poultice made of roasted Onions softens and ripens boils and tumors. Onion soup is used by the French and other nations as a restorative after fatigue, mental depression or want of sleep. Sir John Sinclair thought that there was more nourishment in this, than in any other vegetable; and states, as a well known fact, that a "Highlander, with a very few raw Onions in his pocket and a crust of bread, can do an incredible amount of work, or travel constantly for two or three days together, without any other food."

It is very certain that the Onion was much esteemed; making even slavery bearable, in the days of the Bible, for with the "Promised Land" in view, the Israelites had regretful longings for the "Leech and Onions" of Egypt.

Mash roasted Onion, spread it on a cloth, and pour upon it lard or sweet oil, apply to the throat as hot as can be borne, with a dry and larger flannel covering the edges of the poultice, so as to keep the heat in, let the poultice cover all the throat and upper part of chest for croup or bronchitis; give also a teaspoon of Syrup of Ipecac every 20 minutes.

